# Chronic anterior talofibular ligament rupture is associated with bilateral knee alterations and reduced ankle plantar flexor moment during gait

**DOI:** 10.3389/fspor.2026.1834260

**Published:** 2026-05-18

**Authors:** Liufeng Xiao, Peiyao Liang, Lili Hu, Lin Guo, Lihang Zhang, Chengke Zhang, Molong Chen, Lisha Xu, Shikun Wen, Dianwei Li, Lin Ma

**Affiliations:** 1 Department of Orthopedics/Sports Medicine Center, Southwest Hospital, Army Medical University, Chongqing, China; 2 Department of Public Health, and Department of National Clinical Research Center for Child Health, The Children’s Hospital, Zhejiang University School of Medicine, Hangzhou, China

**Keywords:** chronic anterior talofibular ligament insufficiency, gait analysis, joint kinetics, kinematic alteration, lower extremity biomechanics

## Abstract

**Background:**

With the increasing clinical burden of ankle injuries, particularly anterior talofibular ligament (ATFL) rupture, understanding their biomechanical impact on gait has gained renewed importance. While previous research has established local ankle deficits, the specific proximal compensatory mechanisms—particularly at the knee joint—in patients with MRI-confirmed complete ligamentous discontinuity remain insufficiently characterized.

**Purpose:**

The objective of this study was to investigate spatiotemporal and kinetic alterations in ankle and knee biomechanics in individuals with chronic ligament insufficiency during walking compared to normal controls.

**Methods:**

A total of 78 participants were enrolled in Southwest Hospital. The injury group consisted of 39 individuals with MRI-confirmed complete ATFL rupture scheduled for surgery, recruited from a specialized sports medicine clinic. They were matched with 39 healthy controls. Three-dimensional motion capture synchronized with force plates was used to assess gait parameters, including step/stance times, ground reaction forces, joint angles, and moments.

**Results:**

Patients with ATFL rupture exhibited significantly reduced stride length compared to controls (*p* < 0.05), whereas cadence, walking speed, step width, and the symmetry indices of step time, stance phase, and swing phase did not differ significantly between groups (all *p* > 0.05). Sagittal plane kinematic analysis revealed significantly decreased dorsiflexion and plantarflexion excursions at the affected ankle, leading to reduced plantar flexor power generation. Compensatory mechanisms were observed at the knee, where the affected limb demonstrated significantly decreased flexion angles and extension moments.

**Conclusion:**

Individuals with chronic ATFL rupture exhibit persistent gait asymmetries and altered lower-limb biomechanics, including reduced stride length, diminished ankle plantar flexor moment, and modified kinematics at both the injured and contralateral knee joints. These findings suggest that functional deficits may extend beyond the ankle to affect proximal joints along the kinetic chain. Although the cross-sectional design precludes causal inference, the observed bilateral kinematic alterations at the knee suggest that proximal joint adaptations may accompany chronic ATFL rupture and warrant further investigation in prospective and interventional studies.

## Introduction

Lateral ankle ligament complex sprains constitute a pervasive clinical challenge, representing 10%–30% of all sports-related injuries (1–3). Despite their high prevalence, treatment outcomes remain suboptimal, with recurrence rates reported as high as 73% (4), imposing a significant burden on both individuals and healthcare systems. Within the lateral ligament complex, the anterior talofibular ligament (ATFL) is the most vulnerable structure (5–7). While the calcaneofibular ligament (CFL) acts as a secondary stabilizer, the ATFL is typically the first to fail during inversion stress. As the primary restraint against anterior talar displacement and plantarflexion-inversion stress, the ATFL plays a pivotal role in maintaining ankle joint stability (8, 9). While many acute sprains resolve with conservative management, a significant subset of patients progresses to chronic pathology. Notably, complete ATFL rupture results in distinct pathological mechanical laxity, which differs fundamentally from functional instability often attributed solely to proprioceptive deficits. This loss of structural integrity fundamentally alters the congruency of the talocrural joint during weight-bearing activities (5, 9).

The biomechanical consequences of ATFL insufficiency extend well beyond the local ankle joint. During the normal gait cycle, the lower limb functions as a closed kinetic chain, where forces and motion are transferred sequentially from the foot to proximal segments (10). When the ATFL is incompetent, the ankle's capacity for stabilization during weight acceptance and power generation during push-off is compromised (11, 12). Theoretical models suggest that the locomotor system may compensate for this distal deficit to maintain gait stability and velocity. This compensatory effect may propagate up the kinetic chain, potentially requiring the ipsilateral knee or hip to adopt altered loading strategies (13, 14). As the pathology becomes chronic, these adaptive gait patterns may result in stress concentration at adjacent joints (15, 16).

However, the specific compensatory mechanisms employed by patients with chronic, structural ATFL rupture remain insufficiently characterized. Existing literature has predominantly focused on “Chronic Ankle Instability (CAI)” as a broad category (2), often conflating subjects with functional instability and those with gross mechanical laxity (17). Furthermore, while ankle-level kinematics are well-documented (18, 19), few studies have simultaneously quantified the kinetic alterations at the knee joint based on strict MRI-confirmed diagnoses. Understanding these proximal adaptations is critical, as the link between local ligament injury and global musculoskeletal sequelae remains obscure in current biomechanical models. Clarifying these complex interactions is indispensable for providing the evidence base for transitioning from isolated ankle stabilization to integrated kinetic chain rehabilitation (20, 21).

Therefore, the aim of this study was to characterize spatiotemporal and kinetic alterations in ankle and knee biomechanics during walking in individuals with MRI-confirmed chronic ATFL rupture. By restricting enrollment to patients with complete ligamentous discontinuity, we sought to reduce the clinical heterogeneity commonly present in broader CAI cohorts.

We hypothesize that individuals with chronic complete ATFL rupture will demonstrate altered lower-limb gait biomechanics compared with healthy controls, characterized by (1) distinct time-varying patterns of ankle and knee kinematics and kinetics across the gait cycle, and (2) greater inter-limb asymmetry in spatiotemporal symmetry indices.

## Methods

All experimental procedures were conducted in accordance with the Declaration of Helsinki and approved by the Institutional Review Board of Southwest Hospital (Ethical Approval No. KY2025281). Written informed consent was obtained from all participants prior to data collection, with particular attention given to ensuring comprehension of experimental protocols and potential risks.

### Sample size calculation

An *a priori* power analysis was conducted using G*Power (Version 3.1; Heinrich Heine University, Düsseldorf, Germany). We based our effect size estimation on the systematic review and meta-analysis by Moisan et al. (11), which reported moderate-to-large standardized mean differences (Hedges’ g ranging approx. 0.5–0.8) for sagittal and frontal plane ankle biomechanics in patients with CAI during walking. Consequently, a conservative medium effect size of 0.65 was selected. To achieve 80% statistical power (1−*β*) = 0.80, *β* = 0.20; *α* = 0.05(two-tailed) using a two-tailed independent *t*-test, a minimum of 38 participants per group was required. Accordingly, we recruited a total of 78 participants (39 per group) to ensure robust statistical power.

### Participants

A total of 78 participants were enrolled in this study, recruited from the Sports Medicine Center of Southwest Hospital. They were divided into two groups: the Injury Group (*n* = 39) and the Normal Group (*n* = 39).

#### Injury group

**Figure 1:**
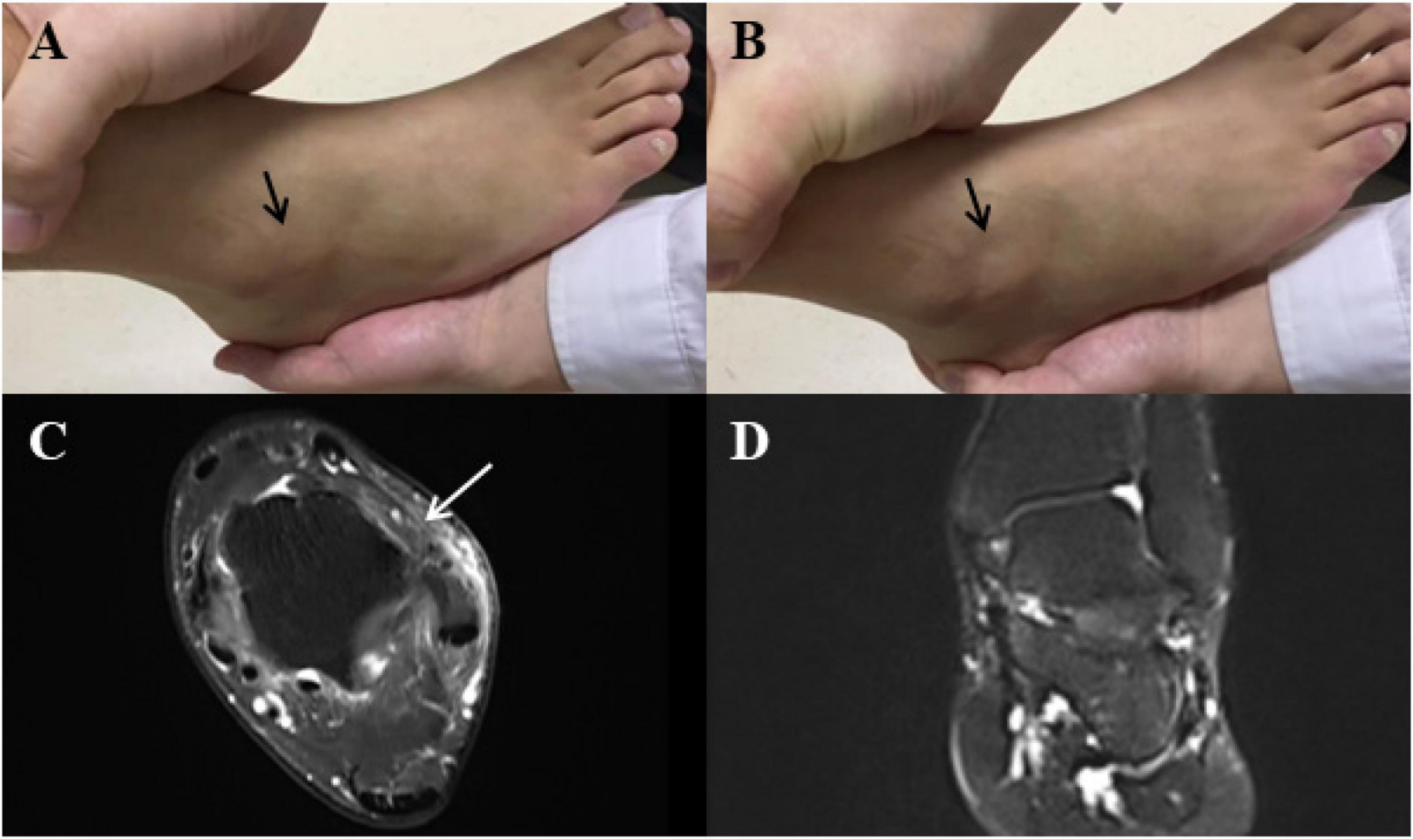
**(A)** before the anterior drawer test of the ankle, the area in front of the lateral malleolus near the anterior talofibular ligament is filled. **(B)** During the anterior drawer test, the talus moves forward, and the area where the anterior talofibular ligament runs becomes depressed (black arrow). **(C)** T2-weighted MRI of the ankle revealed a rupture in the anterior talofibular ligament (white arrow). **(D)** T2-weighted MRI of the ankle revealed no evidence of damage to the articular cartilage.

This group consisted of 39 patients (3 females, 36 males) diagnosed with chronic complete ATFL rupture (≥3 months post-injury). Diagnosis was confirmed via both physical examination (positive Anterior Drawer Test) and Magnetic Resonance Imaging (MRI). Detailed morphological evaluation of the MRI scans revealed that 25 patients (64.1%) presented with an isolated complete rupture of the ATFL. The remaining 14 patients (35.9%) exhibited complete ATFL rupture accompanied by inflammatory signal changes (edema) in the CFL, yet with preserved structural continuity (no rupture). Therefore, the cohort was considered to predominantly represent ATFL insufficiency, with no MRI evidence of CFL rupture. The distinction between CFL edema and CFL rupture carries direct mechanical relevance. Edema without structural discontinuity preserves the gross load-bearing capacity of the ligament, whereas complete rupture fundamentally alters subtalar kinematics under dynamic loading (6, 22). On this basis, classifying the present cohort as representing ATFL insufficiency with intact CFL structural continuity is biomechanically justified. It should be noted, however, that MRI-confirmed structural discontinuity identifies ligament rupture but does not directly quantify *in-vivo* mechanical laxity during dynamic gait tasks; references to ligament insufficiency throughout this study therefore reflect inferred functional implications of structural findings rather than directly measured dynamic instability. Representative MRI characteristics are shown in Figure 1. Pain intensity during walking was assessed using a 100 mm Visual Analogue Scale (VAS; 0 = no pain, 10 = worst imaginable pain), and functional ankle instability severity was quantified using the Cumberland Ankle Instability Tool (CAIT; range 0–30, with scores ≤24 indicating chronic ankle instability).

#### Normal group

The normal group consisted of 39 healthy individuals (2 females, 37 males) matched for age and BMI, with no history of lower extremity musculoskeletal injuries or neurological disorders. The inclusion and exclusion criteria for the subjects are listed at the end of the manuscript.

**Inclusion Criteria for ATFL Group:**

1. Age 20–35 years with BMI 18–25 kg/m²
2. Complete ATFL rupture confirmed by: (a) MRI evidence of full-thickness ligament discontinuity (coronal T2-weighted sequences); (b) The anterior ankle drawer test was positive. Consensus diagnosis by two independent sports medicine physicians (See Figure 1).
3. The patient presents symptoms of pain in the region where the ATFL is located or exhibits signs of ankle instability. Additionally, the patient has experienced these symptoms for a post-injury duration of more than three months, during which conservative management has been unsuccessful.
4. The patient was scheduled for anatomical ATFL repair, and during the procedure, a rupture of the ATFL was confirmed
5. Berndt-Harty osteochondral lesion grade ≤II on MRI
6. No concomitant lower extremity joint pathology

**Exclusion Criteria for ATFL Group:**

1. History of Corticosteroid Injections (past 6 months)
2. NSAID use within 24 h preceding testing
3. Acute pain (VAS ≥5/10 during weight-bearing)
4. Lower limb proprioceptive training (past 6 months)
5. Structural abnormalities: (a) Limb length discrepancy >1 cm (radiographic scalogram); (b) Foot Posture Index (FPI-6) <0 or >6 indicating severe pes planus/cavus
6. Neurological disorders affecting gait
7. Concurrent foot pathologies (e.g., plantar fasciitis, hallux valgus)

**Normal Group Requirements:**

1. Foot and Ankle Ability Measure (FAAM) scores: (a) Activities of Daily Living (ADL) subscale ≥90/100; (b) Sports subscale ≥80/100
2. Normal ankle laxity (anterior drawer test negative)
3. Tegner activity level matched to ATFL group (±1 level)
4. No history of lower extremity injury in the past three years
5. Absence of foot deformities
6. Willingness to participate in gait testing
7. BMI between 18 and 25 kg/m²

### Procedures

Gait analysis was conducted at the Gait Analysis Laboratory of Southwest Hospital Sports Medicine Center. Prior to testing, anthropometric measurements including height, weight, and body mass index (BMI) were recorded for all participants.

**Figure 2:**
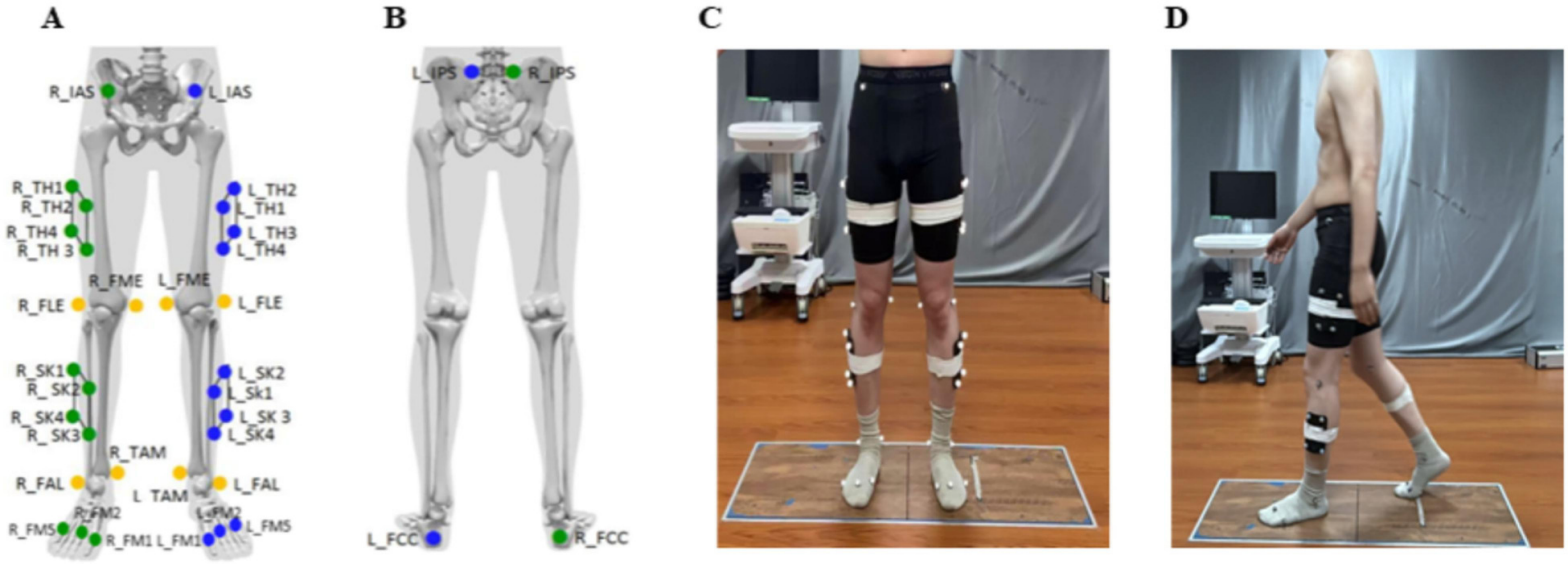
Marker placement of the CAST lower limb model and diagram of gait test. **(A)** Anterior view **(B)** Posterior view **(C)** Test diagram **(D)** diagram of gait test.

Three-dimensional kinematic data were captured using a 10-camera optical motion capture system (Qualisys Arqus A5, Sweden; software version 2022.3.1) synchronized with two force platforms (BMS400600-2K, Advanced Mechanical Technology Inc., USA). Motion trajectories were sampled at 120 Hz and ground reaction force (GRF) signals at 1,200 Hz. Following the CAST (Calibrated Anatomical System Technique) lower-limb model, 28 retroreflective markers were placed on anatomical landmarks during socks-only testing conditions (without shoes) (Figure 2). A static calibration trial was collected to define subject-specific anatomical coordinate systems; during this static trial, additional markers were placed on the medial and lateral femoral epicondyles to identify the knee flexion–extension axis. These epicondyle markers were removed for the subsequent dynamic walking trials to minimize soft-tissue artefact, and the knee joint coordinate system defined during the static trial was applied to all dynamic trials. To minimize inter-rater error, all markers were placed by the same senior research physiotherapist with over five years of experience in clinical gait analysis.

Prior to each data collection session, system calibration was performed using the Qualisys integrated calibration system, maintaining an environmental residual below 0.4 mm. During static calibration, participants stood upright over the force platforms (one foot on each) with arms abducted to 90° to define the anatomical reference frame and joint centers. All markers were placed by a certified clinician, and room temperature was controlled at 22 ± 1°C.

**Figure 3:**
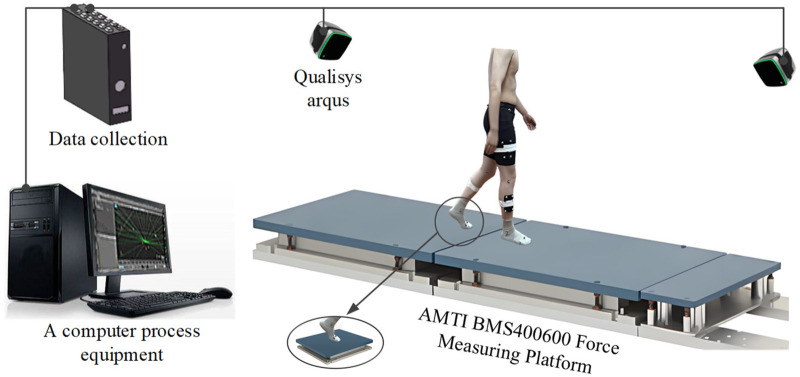
The experimental equipment diagrams.

Each participant completed a familiarization phase consisting of five walking trials at a self-selected speed along a 7 m walkway (see Figure 3). To ensure steady-state walking at the moment of force-plate contact, the force plates were positioned in the middle section of the walkway, allowing each participant to take at least three to four natural steps before the target strike. During the trials, participants were not instructed to target the plates; instead, the starting position was iteratively adjusted so that consistent, single-foot strikes occurred naturally on each plate. Only trials with clean, isolated foot contacts were retained for analysis. Medial and lateral femoral epicondyle markers were used in the static calibration trial only and removed during dynamic trials to reduce soft-tissue artefact. Real-time kinematic monitoring allowed immediate retrials in cases of marker loss, ensuring data quality throughout the session.

### Data processing

Biomechanical data processing was performed in Visual3D (v6.01, C-Motion Inc., USA) using standardized protocols. Raw kinematic and kinetic data were filtered with 4th-order zero-phase Butterworth low-pass filters at 10 Hz and 50 Hz, respectively, based on residual analysis (23). Three-dimensional joint angles were calculated via Cardan/Euler sequences (x-y-z rotation order), defining ankle kinematics as (+) dorsiflexion/inversion/internal rotation and knee kinematics as (+) flexion/adduction/internal rotation using the right-hand rule. Inverse dynamics was performed to compute the net joint moments (*N*·m/kg) and joint powers (W/kg), which were normalized to body mass. These calculations took into account the segmental masses, ground reaction forces, and angular kinematics. The moment of inertia and the center of mass for each segment were determined using the default anthropometric parameters provided in Visual 3D (24). Spatiotemporal parameters were derived from 10 valid gait cycles per participant: gait speed (stride length/time, cm/s), step width (inter-heel mediolateral distance, cm), stride length (distance between two successive heel strikes of the same limb, cm), cadence (steps/min), and phase durations (stance: heel strike-to-toe-off; swing: toe-off-to-heel strike; double support: bilateral contact; all as % gait cycle). Symmetry index (SI) was used to quantify inter-limb differences for each gait parameter using a normalized, unit-free percentage metric derived from the two limbs. SI was computed using affected vs. unaffected limbs in the injury group and dominant vs. non-dominant limbs in controls.

$$\mathrm{SI}\text{\%}=\frac{\left|X_{\mathrm{affected}}-X_{\mathrm{unaffected}}\right|}{0.5*(\left|X_{\mathrm{affected}}|+|X_{\mathrm{unaffected}}\right|)}*100$$

### Statistical analysis

All statistical analyses were performed with a two-sided significance threshold of *α* = 0.05. Data are presented as mean ± SD for normally distributed variables or as median (Q1, Q3) for non-normally distributed variables. Normality was assessed using the Shapiro–Wilk test. SPM waveform analyses constituted the primary outcomes of this study; discrete spatiotemporal parameters were treated as secondary outcomes and were not corrected for multiple comparisons.

Discrete outcome analyses (Tables 2, 3) were performed in GraphPad Prism (version 9.5.0). Normally distributed variables were compared using independent-samples *t*-tests; non-normally distributed variables were compared using Mann–Whitney *U*-tests. Effect sizes are reported as Cohen's d (*t*-tests) or rank-biserial r (Mann–Whitney). Waveform analyses were performed using Statistical Parametric Mapping (SPM) in R (version 4.3.1; spm1d package (25, 26)) on gait waveforms normalized to 101 data points. SPM evaluates the entire continuous time-series simultaneously, exploiting temporal autocorrelation to control family-wise error across time. Paired-sample SPM{t} tests compared the injured vs. uninjured limb within the ATFL group; independent-sample SPM{t} tests compared each limb to the dominant limb of healthy controls. The dominant limb was selected as the reference because it represents the functionally preferred side in healthy individuals, providing a consistent biomechanical benchmark for between-group comparisons. Holm–Bonferroni correction was applied separately for ankle waveforms and knee waveforms, each constituting an independent correction family of six comparisons (three limb-pair contrasts×two variables: moment and angle). The six ankle comparisons were: (1) injured vs. uninjured—ankle moment; (2) injured vs. uninjured—ankle angle; (3) injured vs. control dominant—ankle moment; (4) injured vs. control dominant—ankle angle; (5) uninjured vs. control dominant—ankle moment; (6) uninjured vs. control dominant—ankle angle. An identical family structure was applied to knee moment and knee angle waveforms. The percentage of the gait cycle exhibiting a significant cluster serves as a functional indicator of the temporal extent of group differences; discrete analyses provide complementary descriptive context for overall spatiotemporal performance.

## Results

As shown in Table 1 detailing participant characteristics, seventy-eight subjects participated in this study (39 with ATFL rupture and 39 normal controls). Comparative analysis revealed no significant differences in age, height, weight, BMI (all n.s.), confirming successful group matching for these baseline parameters.

**Table 1:** Basic subject information.

Group	Injury group(*N* = 39)	Normal group(*N* = 39)	*P*
Age (yrs)	26.10 ± 5.09	25.45 ± 4.87	n.s.
Height (m)	1.74 ± 0.06	1.75 ± 0.06	n.s.
BMI (kg/m2)	24.15 ± 2.60	23.55 ± 2.60	n.s.
Symptom duration (months)	7.10 ± 2.46 (4–11)		
VAS during walking	1.59 ± 0.97 (1–3)		
CAIT score	18.0 ± 1.65 (14–22)		

Data are presented as mean ± SD. VAS, Visual Analogue Scale (0 = no pain, 10 = worst pain); CAIT, Cumberland Ankle Instability Tool (0 = maximum instability, 30 = no instability; scores ≤24 indicate chronic ankle instability). VAS and CAIT were assessed in the injury group only.

Analysis of spatiotemporal parameters (Table 2) showed that stride length was significantly shorter in the injury group compared with controls [132.40 (127.20, 139.00) vs. 146.30 (123.30, 153.90) cm, *p* = 0.02]. Cadence did not differ significantly between groups [103.3 (97.44, 107.4) vs. 96.31 (94.24, 118.0) steps/min, *p* = 0.25], and step width remained comparable (*p* = 0.92).

**Table 2:** The spatial-temporal gait parameters between groups.

Group	Injury group (*N* = 39)	Normal group (*N* = 39)	Effect size *(r or Cohen's d)*	*P*
Cadence (steps/min)	103.3 (97.44,107.4)	96.31 (94.24,118.0)	*r* = 0.13	0.25
Speed (cm/s)	112.50 (103.30,124.50)	119.10 (117.10,121.20)	*r* = 0.21	0.07
Stride length (cm)	132.40 (127.20,139.00)	146.30 (123.30,153.90)	*r* = 0.27	**0**.02*
Stride width (cm)	11.41 ± 2.61	11.33 ± 2.72	*d* = 0.03	0.92

Data are presented as mean ± SD for variables that met the normality assumption, and as median (Q1, Q3) for variables with non-normal distributions. Between-group comparisons were performed using independent-samples t tests for normally distributed variables and Mann–Whitney *U*-tests for non-normally distributed variables. All tests were two-tailed, with statistical significance set at *P* < 0.05.

*p < 0.05.

Gait symmetry comparisons between groups (Table 3) indicated greater asymmetry in stride length SI in the ATFL injury group (*p* = 0.04). No significant between-group differences were observed for the SI of step time, stance phase, or swing phase duration (all *p* > 0.05).

**Table 3:** Gait symmetry indices between groups.

Group	Injury group (*N* = 39)	Normal group (*N* = 39)	Effect size	*P*	HL diff (C−I), 95% CI (Lower/Upper)
Stride length (%)	9.41 (6.69,11.23)	8.27 (1.59,10.27)	*r* = 0.23	**0**.04*	−4.78/−0.08
Step time (%)	9.59 (5.86,11.83)	8.50 (4.84,11.47)	*r* = 0.16	0.16	−2.57/0.46
Stance time (%)	5.27 (2.92,7.41)	4.22 (2.08,6.72)	*r* = 0.17	0.12	−2.00/0.19
Swing time (%)	4.39 (2.81,6.44)	4.31 (2.62,6.31)	*r* = 0.00	0.97	−1.01/0.95

Data are presented as median (Q1, Q3). Between-group comparisons were performed using the Mann–Whitney *U*-test. The between-group effect is reported as the Hodges–Lehmann (HL) median difference with its 95% confidence interval (CI). HL difference was defined as Control−Injury (negative values indicate larger SI in the Injury group). Effect size *r* was calculated as *r* = Z/√N, where N is the total sample size across groups. A two-sided *p* < 0.05 was considered statistically significant.

*p < 0.05.

**Figure 4:**
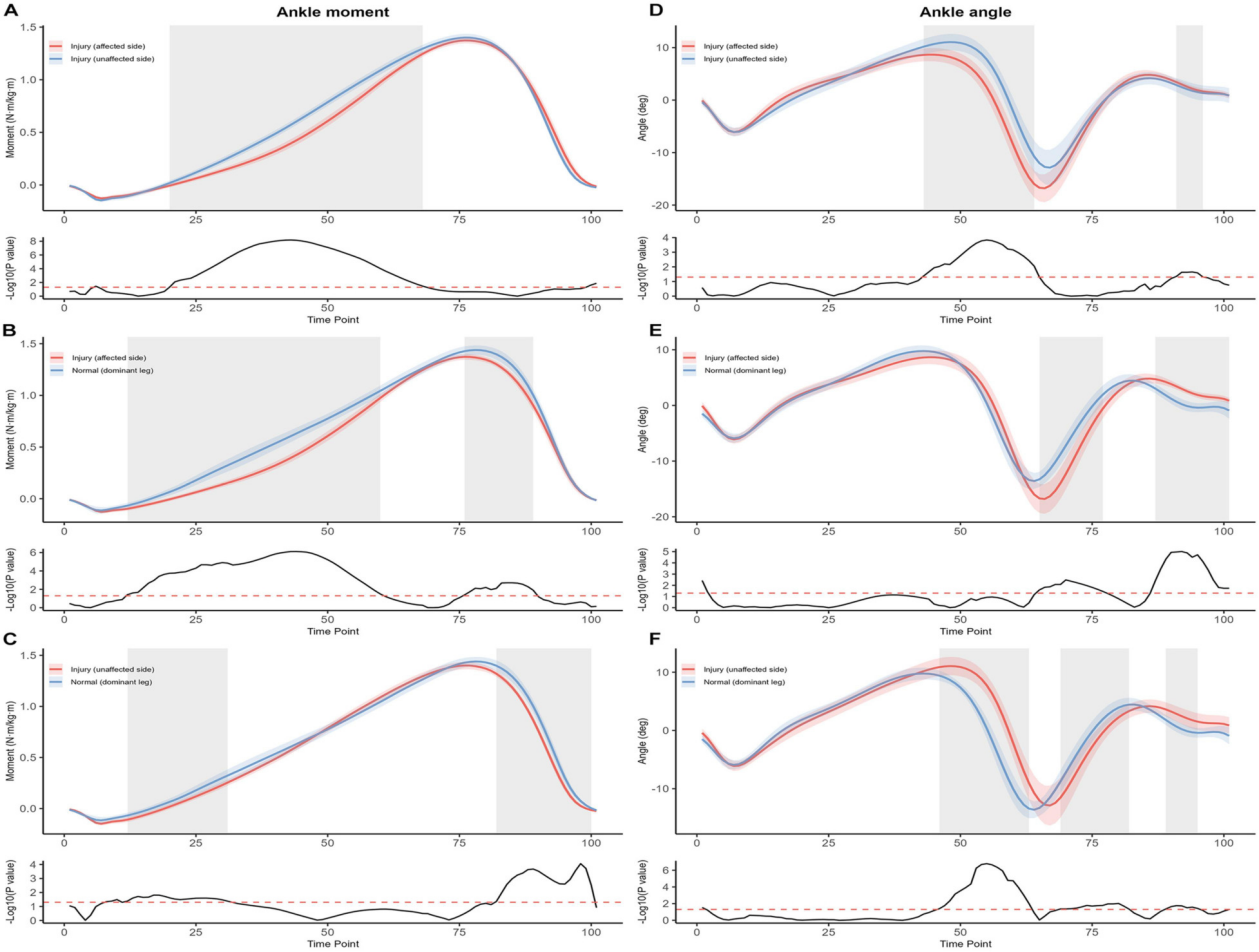
Sagittal-plane ankle kinetics [moment, left column: panels **(A–C)**] and kinematics [angle, right column: panels **(D–F)**] across the normalized gait cycle (0%–100%; heel strike = 0%, toe-off ≈ 60%). For each panel, the upper sub-panel displays group mean waveforms (solid lines) with ±1 SD variability bands (shaded); the lower sub-panel shows the corresponding SPM{t} statistical trajectory with the critical threshold (red dashed line). Panels **(A, D)** injured (affected) limb (red) vs. injured (unaffected) limb (blue) within the ATFL group. Panels **(B, E)** injured (affected) limb (red) vs. dominant limb of healthy controls (blue). Panels **(C, F)** injured (unaffected) limb (red) vs. dominant limb of healthy controls (blue). Sign convention: ankle angle positive = dorsiflexion; ankle moment positive = plantarflexion moment. Highlighted grey bars denote temporal clusters where the SPM{t} statistic exceeded the critical threshold, indicating statistically significant between-group differences (*p* < 0.05 after Holm–Bonferroni correction applied across the six waveform comparisons). The exact gait-cycle percentages and *p*-values for each cluster are reported in the Results.

Statistical parametric mapping revealed distinct alterations in ankle joint mechanics across comparisons (see Figure 4). For the injured vs. uninjured limb (Panels A and D), the ankle plantarflexion moment was significantly reduced on the injured side during mid-to-late stance (20%–68% of the gait cycle, *p* < 0.001). Ankle angle comparison revealed significant deviations in two phases: reduced dorsiflexion during terminal stance (43%–64%, *p* < 0.001) and increased plantarflexion during terminal-swing (91%–96%, *p* = 0.04), suggesting an altered rocker function and push-off strategy in the affected limb. In the comparison between the injured limb and the dominant leg of normal controls (Panels B and E), significant reductions in ankle plantarflexion moment were observed from early stance through terminal stance (12%–60%, *p* < 0.001;76%–89%, *p* < 0.001), indicating diminished force generation capacity. Additionally, the ankle angle on the injured limb demonstrated significant deviations during mid-stance and terminal stance phases (65%–77%, *p* = 0.007; 87%–100%, *p* < 0.001), with increased plantarflexion angles likely reflecting compensatory mechanisms or neuromuscular impairment. When comparing the uninjured limb to the dominant leg of controls (Panels C and F), ankle moments were significantly lower on the uninjured side during early to late stance (12%–31%, *p* = 0.01; 82%–100%, *p* < 0.001). The ankle angle also differed significantly at two time intervals (46%–63%, *p* < 0.001; 89%–95%, *p* = 0.01), with the uninjured limb exhibiting reduced dorsiflexion followed by increased plantarflexion, resembling adaptive changes secondary to contralateral injury.

**Figure 5:**
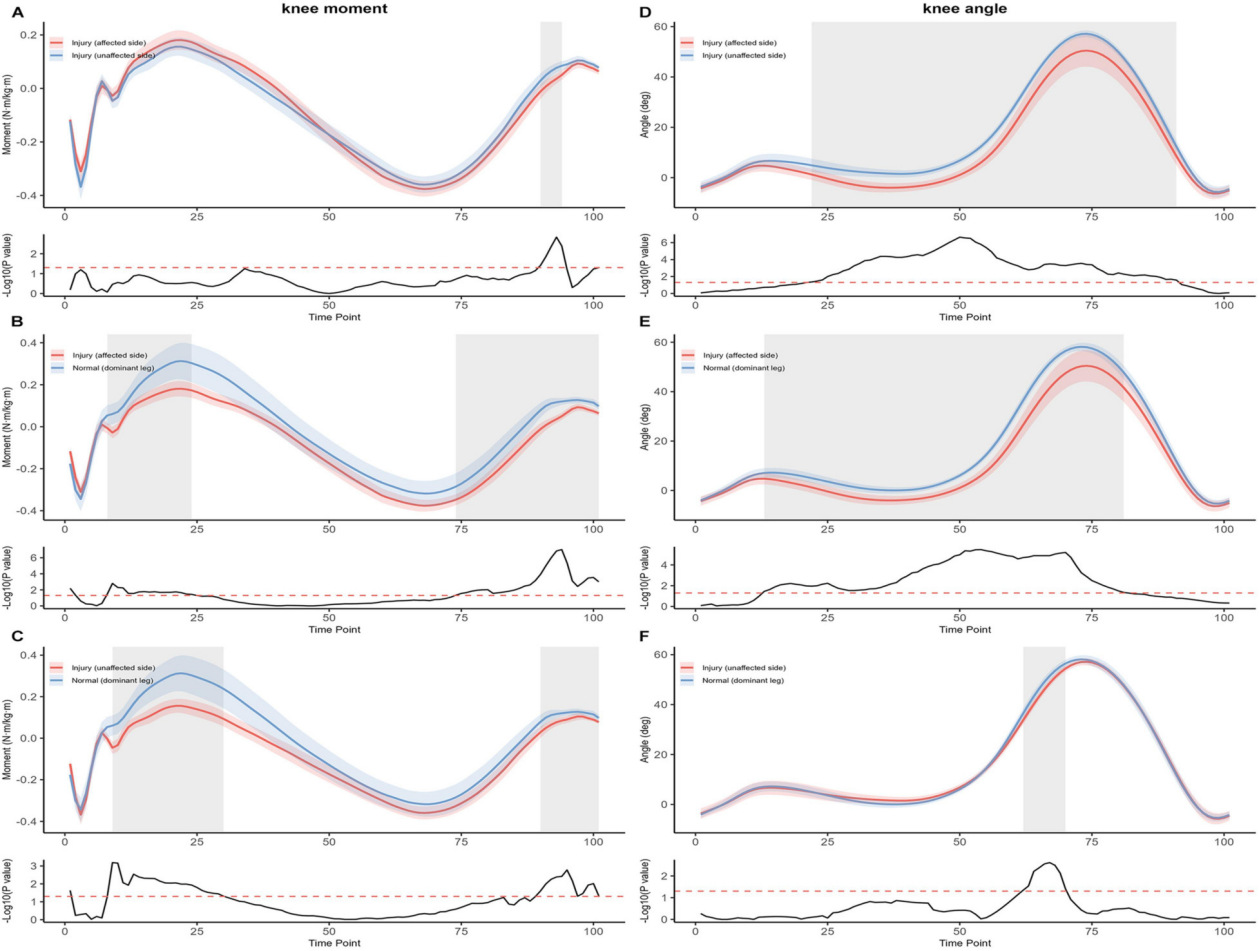
Sagittal-plane knee kinetics [moment, left column: panels **(A–C)**] and kinematics [angle, right column: panels **(D–F)**] across the normalized gait cycle (0%–100%; heel strike = 0%, toe-off ≈ 60%). For each panel, the upper sub-panel displays group mean waveforms (solid lines) with ±1 SD variability bands (shaded); the lower sub-panel shows the corresponding SPM{t} statistical trajectory with the critical threshold (red dashed line). Panels **(A, D)** injured (affected) limb (red) vs. injured (unaffected) limb (blue) within the ATFL group. Panels **(B, E)** injured (affected) limb (red) vs. dominant limb of healthy controls (blue). Panels **(C, F)** injured (unaffected) limb (red) vs. dominant limb of healthy controls (blue). Sign convention: knee angle positive = flexion; knee moment positive = extension moment. Highlighted grey bars denote temporal clusters where the SPM{t} statistic exceeded the critical threshold, indicating statistically significant between-group differences (*p* < 0.05 after Holm–Bonferroni correction applied across the six waveform comparisons). The exact gait-cycle percentages and *p*-values for each cluster are reported in the Results.

In the knee joint SPM analysis (Figure 5), the injured limb showed a significantly reduced extension moment compared with the uninjured limb during terminal stance (90%–94% GC, *p* = 0.007) and a lower flexion angle from mid-stance to terminal swing (22%–91%, *p* < 0.001). Compared with the dominant limb of controls, the injured limb also demonstrated reduced extension moments in early stance (8%–24%, *p* = 0.002) and mid-to-terminal swing (74%–100%, *p* < 0.001), accompanied by lower flexion angle during mid-stance to pre-swing (20%–85%, *p* < 0.001). For the uninjured limb, extension moments were significantly lower than controls during early and terminal stance (9%–30%, *p* < 0.001; 90%–100%, *p* < 0.001), with a slight reduction in knee flexion angle in mid-to-late stance (62%–70%, *p* = 0.02).

## Discussion

The most important finding of this study was that patients with chronic ATFL rupture exhibited bilateral kinematic and kinetic alterations in the lower limb, alongside specific spatiotemporal changes. In the spatiotemporal domain, stride length was significantly shorter in the injury group compared with normal controls, whereas cadence and walking speed did not show statistically significant between-group differences. Waveform analyses further demonstrated reduced ankle plantarflexion moments and altered knee joint mechanics, which likely reflect protective or stabilizing strategies during weight-bearing gait.

In the sagittal plane, a consistent reduction in ankle plantarflexion moment was observed during terminal stance to pre-swing, suggesting diminished push-off capability and impaired distal power generation. Concomitantly, reduced dorsiflexion angles during propulsion were identified, indicating restricted ankle range or protective avoidance of end-range motion. These adaptations may represent neuromuscular strategies aimed at minimizing mechanical instability and pain in the injured ankle.

At the knee joint, the injured limb exhibited a less flexed (more extended) posture and reduced extension torque during early and late stance phases. Such proximal alterations may reflect responses to altered distal ankle mechanics, though a direct compensatory relationship cannot be established from the present cross-sectional data. Rather than increasing torque to overcompensate, the proximal joint appears to adopt a protective strategy to stabilize the lower limb under altered ankle mechanics. Notably, deviations were also found in the contralateral limb relative to healthy controls, suggesting the possibility of bilateral motor adaptations; however, several alternative explanations deserve attention. Contralateral differences could reflect pre-existing inter-limb asymmetry unrelated to the injury, a broader bilateral motor control phenotype, or redistribution of loading strategies adopted to protect the injured limb. The cross-sectional design of this study precludes causal attribution of contralateral changes to the ipsilateral ATFL rupture. Prospective cohort studies with pre-injury baseline measurements are needed to disentangle these possibilities. Given these limitations, the current findings are consistent with prior reports documenting proximal joint alterations in the context of ankle instability (27), and the absence of analogous contralateral deviations in the healthy control group lends tentative support to an injury-related interpretation.

Similar adaptations have been reported in patients with CAI. Specifically, reduced dorsiflexion and altered plantarflexion timing have been demonstrated as mechanisms to maintain joint stability during gait and athletic tasks (28–30). Our findings show a comparable gait pattern, suggesting that ATFL rupture or deficiency may represent an early manifestation of CAI. These collective findings highlight the complex biomechanical adaptations that occur in CAI patients (31), involving both conscious and subconscious neuromuscular adjustments to compensate for ligamentous insufficiency. Consequently, interventions targeting these deficits are crucial; indeed, recent evidence confirms that neuromuscular control training is highly effective in preventing recurrent injuries in this population (32).

A more granular comparison with the CAI literature provides important context for interpreting the present findings. At the ankle level, a systematic review by Moisan et al. (11) identified increased ankle plantarflexion and rearfoot inversion as characteristic gait deviations across 24 CAI studies, broadly consistent with the ankle mechanics observed in our cohort. Herb et al. (33) applied statistical parametric mapping to CAI gait data and demonstrated altered ankle plantarflexion moments throughout the stance phase—the same analytical approach and directionally similar finding as the present study. Regarding proximal and bilateral effects, Ziaei Ziabari et al. (34) demonstrated that unilateral CAI produces compensatory kinematic changes not only at the ipsilateral knee but also at the contralateral knee and hip during gait, while a complementary study by the same group showed that the contralateral ankle in CAI patients exhibits reduced dorsiflexion and increased inversion relative to healthy controls (35). These findings indicate that bilateral biomechanical disruption is a recognized feature of the broader CAI spectrum, lending external support to the contralateral knee alterations documented in the present study.

An important methodological distinction warrants acknowledgment. Most CAI studies use heterogeneous cohorts defined by self-reported instability or clinical stress tests without imaging confirmation of structural integrity. As formalized in the updated instability model of Hertel and Corbett (36), CAI encompasses a spectrum from purely functional instability driven by sensorimotor deficits to mechanical instability defined by complete ligament rupture. Our cohort represents the structural end of this spectrum, confirmed by MRI. Whether gait deficits at this structural extreme are quantitatively more pronounced than those in functional CAI samples remains an open question that future subgroup-stratified research designs should address; the present data do not permit a direct comparison.

The ATFL, as the most vulnerable component of the lateral ankle ligamentous complex, represents the most frequently injured structure in ankle sprains and plays a pivotal role in maintaining ankle joint stability (22, 37). Biomechanically, the ATFL primarily functions to restrain anterior and lateral displacement of the talus, a mechanism critically important during weight-bearing activities including walking, running, and jumping. In individuals with ATFL injury, significant alterations in lower extremity biomechanics are observed, manifesting as gait abnormalities. The gait asymmetries are well characterized in ATFL, including static and dynamic balance assessments. Understanding these deficits is fundamental for diagnosis, rehabilitation, and guiding precise surgical interventions—whether focusing on isolated ATFL repair or combined ATFL and CFL reconstruction—to restore anatomical stability (38, 39).

Emerging research has provided new insights into the biomechanical consequences of ATFL injuries. Caputo et al. (40) demonstrated via quasi-static weight-bearing tests that patients with ATFL deficiency exhibit significant talar internal rotation. Excessive internal rotation of the tibia may alter the normal coupling mechanics of the ankle joint complex, particularly by restricting ankle dorsiflexion. Given the intrinsic coupling between the tibiotalar and subtalar joints, such alterations may induce broader kinematic disturbances across the lower limb (41). These compensatory changes may increase medial tibiotalar cartilage loading, potentially influencing the risk of post-traumatic ankle osteoarthritis. Anatomical variations in the ankle mortise may be risk factors for ankle sprains and may contribute to the early development of osteoarthritis (37). Abnormal ankle alignment may be associated with altered knee joint biomechanics and load distribution, with potential long-term implications for knee joint health that warrant further investigation. Therefore, early assessment of normal joint mobility and joint mechanics in the ankle and knee is crucial to prevent recurrent ankle injuries and osteoarthritis.

The present findings collectively point to a pattern of multi-joint adaptation in chronic ATFL deficiency, with meaningful changes observed not only at the ankle but also at the knee. This broader distribution of biomechanical disruption suggests that rehabilitation targeting the ankle in isolation may be insufficient. The consistently reduced plantar flexor moment at the affected ankle points to triceps surae weakness as a plausible therapeutic target, while the bilateral kinematic shifts at the knee raise the possibility that proximal neuromuscular training—particularly of the knee extensors and hip stabilizers—may complement ankle-directed interventions. These, however, remain clinical hypotheses; whether whole-kinetic-chain rehabilitation translates to better functional outcomes than conventional ankle-focused care is a question that only well-designed randomized trials can answer.

This study has several limitations worth acknowledging. The cross-sectional design means we can describe associations but cannot establish what caused what; longitudinal data would be needed to trace how gait patterns evolve following ATFL rupture. Our sample—predominantly young adult males scheduled for surgery at a single specialist center—likely represents a more severe end of the clinical spectrum, and findings may not extend to women, older individuals, or those managed without surgery. A further consideration is that 35.9% of the injury group showed CFL edema alongside intact structural continuity; whether this influenced mechanoreceptor signaling remains an open question. Lastly, the spatiotemporal comparisons in Tables 2, 3 were exploratory and unadjusted for multiple testing, and no patient-reported functional outcomes were obtained. Additionally, the restricted range of symptom duration in our cohort (4–11 months, spanning only 7 months) limits our ability to examine dose–response relationships between injury chronicity and gait impairment.

## Conclusion

Even in the chronic phase, patients with chronic ATFL rupture exhibit persistent gait asymmetries and altered lower-limb biomechanics. Specifically, the affected ankle demonstrates reduced plantar flexor moment and restricted sagittal plane motion, thereby compromising shock absorption and push-off mechanics. Notably, kinematic and kinetic modifications were observed at both the injured and contralateral knee, suggesting possible bilateral adaptations, although whether these changes reflect injury-related responses or pre-existing asymmetry cannot be determined from the present cross-sectional data.

Taken together, these findings indicate that chronic ATFL rupture is associated with distal ankle deficits and possible bilateral knee adaptations during gait. The clinical relevance of these proximal alterations, and whether they should modify rehabilitation strategies, should be established in future prospective and interventional studies.

## Data Availability

The raw data supporting the conclusions of this article will be made available by the authors, without undue reservation.
